# *Escherichia coli* recombinant expression of SARS-CoV-2 protein fragments

**DOI:** 10.1186/s12934-022-01753-0

**Published:** 2022-02-05

**Authors:** Bailey E. McGuire, Julia E. Mela, Vanessa C. Thompson, Logan R. Cucksey, Claire E. Stevens, Ralph L. McWhinnie, Dirk F. H. Winkler, Steven Pelech, Francis E. Nano

**Affiliations:** 1 Department of Biochemistry and Microbiology, University of Victoria, STN CSC, PO Box 1700, Victoria, BC V8W 2Y2 Canada; 2 Kinexus Bioinformatics Corporation, Vancouver, BC Canada; 3 Division of Neurology, Department of Medicine, University of British Columbia, Vancouver, BC Canada

**Keywords:** SARS-CoV-2, COVID-19, Recombinant spike protein, Carbohydrate-binding module, CBM9, *Escherichia coli*

## Abstract

We have developed a method for the inexpensive, high-level expression of antigenic protein fragments of SARS-CoV-2 proteins in *Escherichia coli*. Our approach uses the thermophilic family 9 carbohydrate-binding module (CBM9) as an N-terminal carrier protein and affinity tag. The CBM9 module was joined to SARS-CoV-2 protein fragments via a flexible proline–threonine linker, which proved to be resistant to *E. coli* proteases. Two CBM9-spike protein fragment fusion proteins and one CBM9-nucleocapsid fragment fusion protein largely resisted protease degradation, while most of the CBM9 fusion proteins were degraded at some site in the SARS-CoV-2 protein fragment. All of the fusion proteins were highly expressed in *E. coli* and the CBM9-ID-H1 fusion protein was shown to yield 122 mg/L of purified product. Three purified CBM9-SARS-CoV-2 fusion proteins were tested and found to bind antibodies directed to the appropriate SARS-CoV-2 antigenic regions. The largest intact CBM9 fusion protein, CBM9-ID-H1, incorporates spike protein amino acids 540–588, which is a conserved region overlapping and C-terminal to the receptor binding domain that is widely recognized by human convalescent sera and contains a putative protective epitope.

**Supplementary Information:**

The online version contains supplementary material available at 10.1186/s12934-022-01753-0.

## Introduction

One of the public health surveillance tools needed to respond to the coronavirus disease 2019 (COVID-19) pandemic is the ability to detect seroconversion to antigens of the SARS-CoV-2 virus, the causative agent of COVID-19. The ability to detect antibodies that are specific to SARS-CoV-2 allows an assessment of the level of probable immunity to COVID-19 in a population. At the individual level, the ability to detect anti-SARS-CoV-2 antibodies can help one assess their personal level of vulnerability to COVID-19 due to immunity generated by either vaccination or from an indeterminate or asymptomatic infection.

The human antibody response to SARS-CoV-2 infection can include the development of antibodies reactive with any of the 29 proteins encoded in the viral genome [1], including 16 non-structural proteins (NSP’s) encoded by the ORF1a/b gene. However most studies have focused on studying the antibody response to the abundant spike and nucleocapsid proteins [2–11]. Among the SARS-CoV-2 proteins, the spike protein varies the most between coronaviruses [12]; using it allows for the greatest specificity in an antibody assay as well as the ability to differentiate reaction with SARS-CoV-2 from other common coronaviruses. As well, some antibodies reactive with the spike protein, especially those that react within or near its receptor binding domain (RBD), are neutralizing [9, 11, 13–15]. Thus, detection of anti-spike protein antibodies may indicate a level of immunity to SARS-CoV-2.

Some studies of anti-SARS-CoV-2 antibody response examine antibody reactivity with linear epitopes using synthetic peptides that correspond to the primary structure of viral proteins [2–10]. While this type of assay misses many antibody responses against conformational and topographically assembled epitopes, it is the most practical, both technically and economically. Although synthetic peptides are far less expensive than full-length recombinant SARS-CoV-2 spike protein, their production cost can still present barriers in resource-poor health systems or when large quantities are needed.

The SARS-CoV-2 spike protein is the most common antigen used to test for seroconversion, and recombinant spike protein, the RBD fragment or synthetic peptides corresponding to spike sequences are commonly used to test sera. Typically, human derived HEK293 cells are used to express spike protein, and Stadlbauer et al. [16] reported expression levels of about 20 mg/L for the RBD fragment and about 5 mg/L of the trimer form of the full-length spike protein. However, other recombinant expression systems have been used to produce the SARS-CoV-2 spike protein. For example, Yang et al. [13] expressed the spike protein RBD in the Sf9 insect cell line (*Spodoptera frugiperda)* using a BAC-to-BAC expression system, and Fujita et al. [17] expressed full-length spike protein in silk worm larvae at a level of about 10 mg/L of larval serum. Recently Rihn et al*.* [18] described the construction of glutathione S-transferase (GST) and maltose binding protein (MBP) fusions to all of the ORFs of SARS-CoV-2, as part of an expansive effort to develop molecular tools to study SARS-CoV-2. These fusion proteins are expressed in *E. coli*, and while their properties and yields are not reported, these recombinants represent significant tools for obtaining SARS-CoV-2 antigenic material. Other groups have expressed the RBD fragment in *E. coli*, though it is largely insoluble and therefore requires solubilization and refolding, perhaps due to the incompatibility between the four disulphide bonds in the native RBD structure and the reducing environment of the *E. coli* cytoplasm [19–21]. As well, the RBD has four confirmed glycans which are absent when the fragment is produced in *E. coli*, leading to differences in antibody binding between human-expressed and *E. coli*-expressed RBD fragments but maintenance of ACE2 receptor binding [19–22]. Maeda and Tian et al. [23] expressed peptides from the spike proteins of the betacoronavirus SARS-CoV-2 and alphacoronavirus porcine epidemic diarrhea virus (PEDV) on the surfaces of genome-reduced *E. coli*, and used those strains as killed whole-cell vaccines for protection against PEDV. The peptide fragments they used, corresponding to SARS-CoV-2 spike protein residues 812–833 and PEDV spike protein residues 884–909, are highly conserved between coronaviruses and vaccination and boosting with both killed whole-cell vaccines provided protection against PEDV. In a similar fashion, our work explores the utility of expressing SARS-CoV-2 peptide epitopes in *E. coli*, using the under-appreciated protein carrier family 9 carbohydrate-binding module (CBM9) from the *Thermotoga maritima* enzyme xylanase 10A [24, 25] that promotes soluble high level protein expression and uses inexpensive reagents for protein purification [26, 27].

## Materials and methods

### Recombinant techniques

**Fig. 1:**
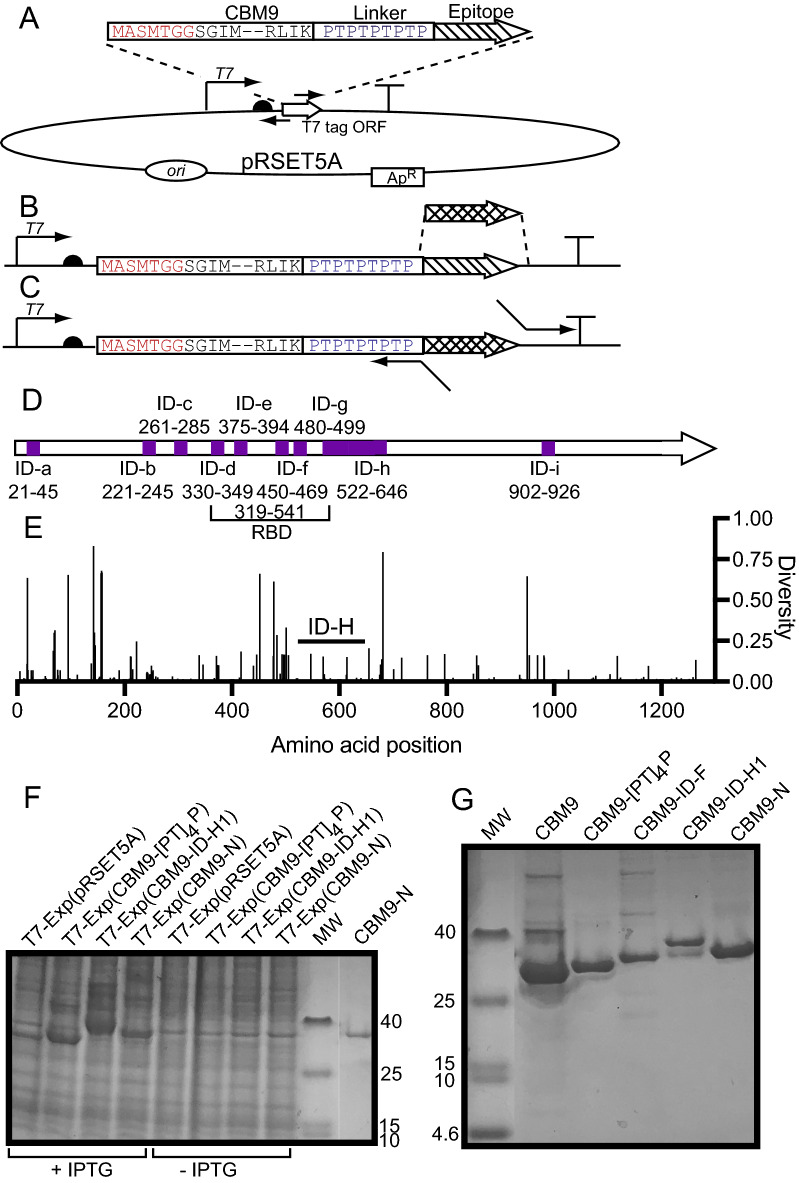
CBM9-SARS-CoV-2 epitope cloning strategies and recombinant fusion protein expression. **A** Initial clones were made by amplifying pRSET5A by inverse PCR, and ligating the plasmid amplicon to synthetic DNA encoding CBM9 with a linker fused to spike protein epitope ID-C, ID-F, or ID-H3. **B** To create fusion clones of ID-A, B, D, E, G, H1 and I, synthetic DNA encoding just the epitope regions replaced the ID-C encoding region. **C** To create the clones CBM9-(PT)_4_P, and N, primers with long overhang regions were used in an inverse PCR reaction using pRSET5A::*CBM9-id-a* to exchange the epitopes fused to CBM9. **D** Representation of linear ID-A through ID-I regions with the amino acid numbers of the SARS-CoV-2 spike protein recognized by antibody from COVID-19 convalescent sera, as described by Zhang et al. [10]; RBD is the receptor binding domain. **E** Diversity of amino acid residues in the SARS-CoV-2 spike protein. Spike protein entropy/diversity data from the Nextstrain SARS-CoV-2 latest global analysis open data was downloaded on January 6th 2022 and plotted using GraphPad Prism 9 [37]. Diversity/entropy values are reported from 0 to 1, where higher numbers represent greater diversity/uncertainty in the amino acid at that position. **F** Cell extracts, equivalent to 33 μL of detergent-soluble material, of recombinants encoding CBM9 fusion proteins with and without IPTG induction. For comparison 1.3 µg of purified CBM9-N is shown in the last lane on the right. **G** Samples of CBM9 fusion proteins purified by batch absorption to cellulose powder, after storage at 4 °C for a minimum of two weeks. In Panels A-C the bent arrow indicates T7 promoter region; half circle indicates T7 ribosome binding site; “T” symbol represents transcriptional terminator

Plasmid pRSET5A was used as the backbone for all expression plasmid constructs [28]. All of the synthetic DNA regions designed to encode CBM9-SARS-CoV-2 spike protein fusions were made by Twist Biosciences. To initially test the expression of CBM9 peptide fusions we cloned synthetic DNA encoding CBM9, CBM9-ID-C, CBM9-ID-F and CBM9-H3 (Fig. 1A). Plasmid pRSET5A was amplified by inverse PCR using primers F-R5A and R-R5A, which have Esp3I sites added to the ends that upon digestion yield 5’-overhangs compatible with the overhangs generated for the PCR amplicons of the synthetic DNA fragments. The CBM9-C, F and H3 DNA fragments were codon optimized [29] for *E. coli* and designed to lack an internal Esp3I site. These fragments were amplified with primers F-CBD (forward primer for all fragments) and R-CBD-IDc, R-CBD-IDf and R-CBD-h3 as the reverse primers. After amplification the products were joined to pRSET5A using a simultaneous cutting and ligation reaction [30] using Esp3I as the restriction enzyme. Briefly, 30 cycles of 5 min at 37 °C and 5 min at 16 °C were followed by 10 min at 65 °C. Ligated DNA was transformed into T7 Express *lysY/I*^*q*^* E. coli* (NEB) and selected on LB agar (per liter, 5 g yeast extract; 10 g tryptone, 5 g NaCl; 15 g agar) supplemented with chloramphenicol (10 μg/mL) and carbenicillin (250 μg/mL). Once initial clones were sequence verified and shown to produce the appropriate protein product, further recombinants were constructed using the pRSET5A::*CBM9-id-c* clone as the backbone. This plasmid was amplified by inverse PCR using primers Fb-R5A and R-R5AidC so as to remove the SARS-CoV-2 spike protein-encoding fragment of DNA and replace it with another fragment of synthetic DNA (ID-a, b, d, g, h, h1, h2, i; Twist Biosciences) (Fig. 1B) using a cutting-ligation reaction as described above. To make a plasmid encoding just the CBM9-(TP)_4_P (no SARS-CoV-2 fragment) or CBM9-N (containing a nucleocapsid epitope), plasmid pRSET5A::*CBM9-id-a* was amplified with primers nF2-R5A-CBD and nR-R5A-Flex; or F-nucl-ep and R-nucl-ep and Esp3I digested and ligated with a strategy depicted in Fig. 1C. The primers used to make all of the constructs are listed in Additional file 1: Table S1. A color-coded example of a CBM9 fusion clone is shown in Additional file 1: Fig. S1. All CBM9-SARS-CoV-2 recombinants expressing protein fusion constructs were sequence verified. The GenBank accession numbers and the availability of recombinant clones is described in Additional file 1: Materials and Methods.

### Fusion protein isolation and analysis

Frozen aliquots of seed cultures were prepared and 0.4 mL of the stocks were added to 20 mL of modified auto-induction ZYM-5052 media that lacks an added carbon source (1% N-Z amine, 1% yeast extract, 25 mM Na_2_HPO_4_, 25 mM KH_2_PO_4_, 50 mM NH_4_Cl, 5 mM Na_2_SO_4_, 2 mM MgSO_4_, 100 µM FeCl_3_) [31]; the media was supplemented with carbenicillin (250 µg/mL) and chloramphenicol (10 µg/mL). These cultures were grown at 24 °C for 16 h. The A_600_ was determined and the culture was added to 100 mL fresh broth with antibiotics in a 1 L flask to give an A_600_ of 2. IPTG was added to a final concentration of 4 mM, and the culture was incubated with shaking (250 RPM) for 3 h at 30 °C. The cultures were cooled on ice for 15 min, and then subjected to centrifugation for 10 min at 16,000×*g*. The cell pellet from each 25 mL of culture was suspended in 5 mL of solution B (500 mM NaCl, 10 mM MgCl_2_, 0.5% CHAPS, 50 mM potassium phosphate, pH 7.0, 100 μg/mL lysozyme). 1.5 g of glass beads (≤ 106 µm, Sigma), and 5 µL Benzonase® nuclease (Sigma) were added and the cell suspension was vortexed vigorously for four 1 min intervals. The beads and insoluble debris were pelleted by centrifugation at 16,000×*g* for 10 min and the supernatant was transferred to a new tube. A second 5 mL of solution B was added to the beads, the vortexing and pelleting steps were repeated and the supernatant was combined with the supernatant from the previous lysis step. The pooled supernatant was added to 2.5 g of cellulose powder (Sigma, cat # 435236) that had previously been equilibrated with solution A (500 mM NaCl, 50 mM potassium phosphate, pH 7.0) in a 50 mL conical centrifuge tube. The tube was rocked for 16 h at room temperature, and the cellulose powder was pelleted by centrifugation for 2 min at 4000×*g*. The cellulose resin was washed three times by adding 12 mL of solution A, rocking for 2 min, and separation of the cellulose from the washing solution. This was followed by three washes using solution C (150 mM NaCl, 50 mM potassium phosphate, pH 7.0). Following the last wash with solution C, the cellulose was suspended in 5 mL elution buffer (1 M glucose, 15 mM NaCl, 10 mM Tris, pH 7.6), and rocked for 30 min. Centrifugation was applied to pellet the cellulose, the supernatant was removed, the elution step was repeated twice more and eluates were pooled. Pooled eluates were added to a Vivaspin® 6, 10 kDa MW cut off (Sartorius) protein concentrator to change the buffer to 150 mM NaCl, 10 mM Tris, pH 7.6.

Protein concentrations of aqueous samples were determined using the Qubit protein assay (ThermoFisher). Following SDS-PAGE separation a LI-COR Odyssey® CLx Imager was used to measure the fluorescence following excitation at 700 nm of Coomassie blue stained protein bands. The laser scan data was processed with Image Studio v5.2 software which was used to determine the relative amount of specific recombinant protein bands to the total protein in a sample.

### Mass spectroscopy analysis of recombinant proteins

Liquid chromatography–mass spectrometry for the determination of intact molecular weight of isolated CBM9 fusion proteins was conducted by the University of Victoria/Genome BC Proteomics Centre. Details of the methods are presented in Additional file 1: Materials and Methods.

### Commercial recombinant proteins and antibodies

The following recombinant SARS-CoV-2 proteins [18] were procured from the MRC Protein Phosphorylation and Ubiquitination Unit Reagents and Services at the University of Dundee (Dundee, Scotland; https://mrcppu-covid.bio/), and are listed in Additional file 1. Rabbit polyclonal antibodies (Kinexus Bioinformatics) directed against synthetic peptides based on SARS-CoV-2 proteins were used in dot blots, and are listed in Additional file 1.

### Human serum samples

The process of the use of human blood products for the investigation of COVID-19 received an ethical approval by Veritas Independent Review Board Inc. (Saint-Laurent, QC, Canada), IRB Protocol 16,567–09:39:354–06-2020. The majority of blood serum samples were generated from blood donations of volunteers. A small number of serum or plasma samples from positive tested donors and all pre-COVID-19 samples were acquired by purchase (Precision for Medicine, Norton, MA, USA; Innovative Research, Novi, MI, USA; AllCells, Alameda, CA, USA) or by donation (CureImmune, Vancouver, BC, Canada).

The blood samples were collected from persons that had been confirmed as positive using a PCR genetic test (designated as “COVID”), those that showed symptoms similar to COVID-19 but were not tested (designated as “sick”), those that were healthy and asymptomatic (designated as “Control”) and those that were healthy and donated prior to April 2019 (“pre-COVID”).

All of the preparations of recombinant proteins were robotically spotted as 0.24 µL of a ~ 8 µM final concentration (except GST-NSP2 SARS CoV2 [DU 66414] in Spot B1 was printed at a 6.5 µM concentration) on to nitrocellulose membranes. The blots were washed three times with TBS (aqueous solution of 20 mM Tris-base and 250 mM NaCl; pH 7.5). These dot blot arrays were blocked with 2.5% BSA in T-TBS (TBS with 0.05% Tween 20) for 30 min. After washing the membranes twice with T-TBS, the arrays were incubated with either affinity-purified rabbit polyclonal antibodies against SARS-CoV-2 protein sequences at 2 µg/mL or serum from recovered COVID-19 patients and healthy controls at 1:200 dilution in T-TBS. The incubation was carried out at 4 °C overnight. To detect the bound antibodies, the arrays were washed with T-TBS three times followed by the incubation with goat anti-human IgG + IgA + IgM pAb or donkey-anti-rabbit IgG (HRP conjugates, 1:20,000 dilution; Jackson ImmunoResearch, West Grove, Pennsylvania, USA). After 30 min incubation, the arrays were washed six times with T-TBS, once with 125 mM NaCl and rinsed twice with water. The bound secondary antibody was visualized by enhanced chemiluminescence (ECL) on a Bio-Rad FluorS-Max scanner. The ECL scan was performed at a scanning time of 300 s. Eight images of the scanned array were generated during that scanning time.

## Results and discussion

### Expression of SARS-CoV-2 protein fragments as fusions to the CBM9 module

The goal of our research was to test if SARS-CoV-2 protein fragments known to elicit a human antibody response could be produced inexpensively using a universally available microbial expression system. The SARS-CoV-2 spike protein or its RBD have been recombinantly produced in a number of hosts, including engineered HEK-293 cells, insect cells, insect larvae and *Escherichia coli* [17, 19–21, 32–36]. However, these eukaryotic expression systems require growth media and bioreactors that are orders of magnitude more expensive than the material and equipment used for microbial expression systems, and *E. coli*-produced RBD fragments are insoluble, and require refolding. Therefore, we felt that there was value in developing an inexpensive microbial expression system that can produce soluble SARS-CoV-2 protein fragments in substantial amounts.

Our strategy was to use a standard *E. coli* T7 RNA polymerase approach to drive high levels of mRNA transcription, and use a proven carrier protein module, a thermophilic family 9 carbohydrate-binding module (CBM9), to carry the SARS-CoV-2 fragment at the C-terminus of the fusion protein (Fig. 1A–C). Kavoosi et al. [26] showed that the CBM9 module expresses at high levels, even when a protein was fused to the C-terminus. In a separate work [27], these same researchers showed that linking CBM9 to a protein with a proline-threonine rich linker ([PT]_4_P-IEGR) resulted in a fusion protein that was resistant to protease attack by endogenous *E. coli* proteases [27]. Thus, we adopted the use of (PT)_4_P as a linker between the CBM9 and SARS-CoV-2 spike protein fragments, and an example of the gene organization is shown in Additional file 1: Fig. S1.

The bulk of the studies on the antibody responses to SARS-CoV-2 have been conducted using overlapping synthetic peptides corresponding to SARS-CoV-2 proteins, primarily the spike protein, but sometimes several proteins or the entire proteome. At the time that we initiated our studies, the work of Zhang et al*.* [10] was one of the more comprehensive analyses of the human antibody response in COVID-19. These researchers identified nine immunodominant amino acid regions in the spike protein, designated ID-A through ID-I (Fig. 1D), which were recognized by antibodies from COVID-19 convalescent sera, and we chose these regions as well as one nucleocapsid protein epitope to clone and express. An SDS-PAGE analysis of the expressed CBM9-spike protein fusions (Additional file 1: Fig. S2) indicated which fusion proteins were most resistant to degradation by *E. coli* and we chose a sub-group of the clones to study further (abandoning ID-H, ID-H2, ID-H3 and ID-I). Table 1 lists the amino acid sequences of the encoded spike (and one nucleocapsid [“N”]) protein regions in the clones we constructed and chose to study further, and the specific epitope regions of the spike protein identified by Zhang and co-workers [10] are underlined. As the COVID-19 pandemic has progressed many amino acid changes have been observed in the SARS-CoV-2 spike protein, and spike protein amino acid diversity data, obtained from Nextstrain.org [37], is shown in Fig. 1E.

**Table 1:** Amino acid sequence of B-cell epitopes fused to CBM9, and putative protease cleavage sites

Epitope (amino acid numbers)	Amino acid sequence and calculated MW of processed protein*, and observed MW of CBM9 fusion protein	
ID-A (spike 21–45)	NLTTRTQLPPAYTNSFTRGVYYPDKVF/RSSVLHSTQ	
	Calculated: 26,076.08	Observed: 26,075.1
ID-B (spike 221–245)	RDLPQGFSALEPLVDLPIGINITR/FQTLLALHRSYLTPGD	
	Calculated: 25,560.93	Observed: 25,559.9
ID-C (spike 261–285)	SSGWTAGAAAY/YVGYLQPRTFLLKYNENGTITDAVD	
	Calculated: 23,967.94	Observed: 23,966.9
ID-D (spike 330–349)	ESIVRFPNITN/LCPFGEVFNATRFASVYAWNRKR	
	Calculated: 24,216.16	Observed: 24,215.2
ID-E (spike 375–394)	NSASFSTFK/CYGVSPTKLNDLCFTNVYAD	
	Calculated: 23,914.95	Observed: 23,913.9
ID-F (spike 450–469)	DSKVGGNYNYLYRLFRKSNLKPFERDISTEIYQ	
	Calculated: 26,940.52	Observed: 26,939.5
ID-G (spike 480–499)	QAGSTPCNGVEGFNCYFPLQ/SYGFQPTNGVGYQ	
	Calculated: 23,967.94	Observed: 23,966.9
ID-H1 (spike 540–588)	NFNFNGLTGTGVLTESNKKFLPFQQFGRDIADTTDAVRDPQTLEILDIT	
	Calculated: 28,384.23	Observed: 28,383.2
N (nucleocapsid 386–403)	QKKQQTVTLLPAADLDDF	
	Calculated: 24,957.55	Observed: 24,956.57
CBM9-(TP)_4_P	Not applicable:	
	Calculated: 22,945.49	Observed: 22,944.51

*The calculated molecular weight was based on sequences assuming that the amino terminal methionine was removed. The C-terminal amino acid was chosen to best match the molecular weight of the dominant form found in the MS experiments; in most cases the C-terminal amino acid lies before the slash (“/”). Underlined sequence corresponds to synthetic peptide found to bind antibody as determined by Zhang et al. [10]. The region surrounding the slash indicates the putative protease cleavage site, by endogenous *E. coli* proteases

### Purification and mass spectroscopy analysis of CBM9 fusion proteins.

From the recombinant CBM9 fusion clones that we chose, we expressed and isolated the recombinant protein using powdered cellulose in a batch purification. The resulting purified proteins were subjected to mass spectroscopy analysis to determine the molecular weight of the dominant purified product. The results (Table 1) indicated, as expected, that all products had the N-terminal methionine removed. Most cloned products were processed, presumably by endogenous *E. coli* proteases, so that some portion of the C-terminal end was removed, effectively removing a few to several amino acids of the spike protein fragment. However, clones expressing CBM9-(PT)_4_P, CBM9-ID-F, CBM9-H1 and CBM9-N produced, as the dominant purified product, proteins that were 1 Dalton smaller than the predicted monoisotopic product. Since the CBM9-(PT)_4_P dominant product was 1 Dalton smaller than predicted, we interpreted this to mean that an unidentified chemical modification occurs, many of which are documented [38], on the CBM9 module to remove one atomic mass unit. The clone expressing CBM9-ID-A was processed so as to remove only two amino acids from the B-cell epitope identified by Zhang et al. [10].

For further work we chose *E. coli* clones that expressed intact fusion proteins of CBM9 and fragments of SARS-CoV-2 proteins as the dominant recombinant products. The expression of clones CBM9-(PT)_4_P, CBM9-ID-H1 and CBM9-N are shown in Fig. 1F and the purified products in Fig. 1G; for comparison, the carrier protein module CBM9 is also shown. By comparing the staining intensity of the protein band in the cell extracts to the band of purified CBM9-N, we estimated that the clones expressed recombinant product at levels of at least 100 mg/L upon IPTG-induction, and this is consistent with the 200 mg/L estimates of Kavoosi et al. [26]. We examined the purification yield of clone CBM9-ID-H1 (Table 2 and Additional file 1: Fig. S3). Because the CBM9-ID-H1 lacks any enzymatic activity that can be used to quantify its specific activity we used the inherent fluorescence of Coomassie blue stained proteins with 700 nm excitation to measure the relative amount of CBM9-ID-H1 in a protein mixture of known protein content. After 6 h of IPTG induction this clone yielded recombinant protein at about 27% of the total soluble protein, or 756 mg/L. The final recombinant protein yield of the specific protein band confirmed by mass spectroscopy to be the complete, intact CBM9-ID-H1 fusion protein was 16%, or 122 mg/L. A slightly smaller protein co-purified with CBM9-ID-H1, and presumably this is a proteolytic fragment of CBM9-ID-H1. This lesser band represents 33% of the total protein of the final purified protein preparation, and, thus, the CBM 9-ID-H1 is about 67% pure. That is, the final purified protein preparation yielded 122 mg/L of CBM9-ID-H1, and 60 mg/L of a fragment of CBM9-ID-H1. The contaminating band could be removed using a further purification step, but this may be unnecessary in an antibody detection assay as the contaminating protein is unlikely to interfere in the assay any more than the CBM9 portion of the CBM9-ID-H1 fusion protein. These experiments were performed using standard research growth flasks at an A_600_ of less than 10, and it is likely that the levels of recombinant protein produced could be significantly increased using an optimized fed-batch bioreactor protocol.

**Table 2:** Purification steps for CBM9-ID-H1 isolation

Purification step	Total protein (mg/L)	CBM9-H1 (mg/L)	Yield (%)	Fold purification
Soluble cell extract	2800	756	100	1
Eluate from cellulose resin	298	242	32	3
After buffer exchange	182	122	16	2.5

Different treatments were tested for their effect on the stability of purified CBM9 fusion protein (Fig. 1G). The CBM9 and the CBM9-ID-F samples were heated to 70 °C for 10 min and the CBM9-(PT)_4_P, CBM9-ID-H1 and CBM9-N samples were filtered sterilized, all before storing at 4 °C for at least two weeks (Fig. 1G). As well, samples were stored at −20 °C in 50% glycerol (Fig. 1F last lane on right, CBM9-N). All storage conditions preserved the integrity of the sample. However, heating to 70 °C seemed to generate small amounts of multimers of the protein, consistent with previously reported observations for hyperthermophilic enzymes [39].

**Fig. 2:**
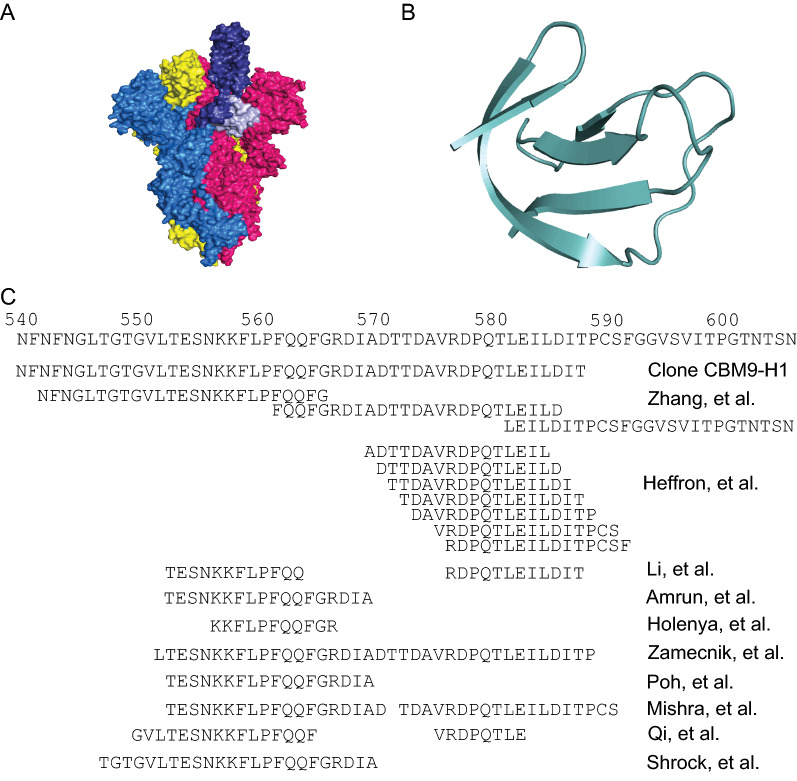
Immunodominant amino acid region “H” of the spike protein. **A** Surface topology view of an electron microscopy structure of the spike protein trimer in the open state (PBD: 6VYB) [41], created with PyMOL [42]. The spike protein subunit in the up position is colored in blue and the subunits in the down position are colored in yellow and pink. The RBD of the blue subunit is shown in dark blue and the H1 region in light blue, though it is important to note that some residues of the RBD are missing in the electron microscopy structure. **B** Cartoon representation of the H1 region of the spike protein (PDB: 6VYB) [41], created with PyMOL [42]. **C** Immunodominant region of the SARS-CoV-2 spike protein. Several groups have identified the region that encompasses approximately amino acids 540–600 as a region that elicits antibody response following infection

We constructed a number of clones of the ID-H region (see Additional file 1: Fig. S2C), and it was fortuitous that the CBM9-ID-H1 clone highly expressed a product that was largely resistant to *E. coli* proteases. The ID-H region (residues 522–646) partially overlaps with the RBD (residues 319–541) of the SARS-CoV-2 spike protein, is only missing one confirmed glycosylation at N616 when produced in *E. coli* [22], and contains no cysteine pairs involved in disulphide bonds (though it does contain C525 which forms a disulphide bond with C391 in the RBD) [20]. In the 3D-structure oriented with the RBDs at the top, the ID-H1 region (residues 540–588) slightly overlaps with and lies below the RBD (Fig. 2A). It is possible that the CBM9-H1 recombinant product is resistant to proteases, while shorter CBM9 fusions are susceptible, because the ID-H1 clone encodes a potential self-folding protein domain (Fig. 2B). The region encompassed by CBM9-ID-H1 includes amino acid sequences identified by several groups as B-cell epitopes, as defined by synthetic peptides that are recognized by convalescent sera from COVID-19 patients (Fig. 2C).

With the exception of the Alpha variant mutation A570D and the Omicron variant mutation T547K, all of the amino acid changes in the spike protein found in SARS-CoV-2 variants of concern lie outside of the region encompassed by the H1 clone [12, 37]. Additionally, the A570D mutation has decreased in frequency over time and is now exclusively an alanine, meaning that there is only one mutation in the H1 region at this time. Globally over time, the ID-H1 region appears to have lower diversity or higher conservation than other regions of the spike protein (Fig. 1E), but due to the variation in diversity at each amino acid position this observation is not statistically significant. As well, Poh et al. [9] found that titer of antibody in sera from COVID-19 convalescent patients that reacted with a peptide corresponding to amino acids 562–579 of the spike protein correlated with the amount of in vitro pseudovirus neutralization. When the neutralizing sera was depleted of reactivity against this peptide the neutralization activity fell sharply. Such evidence indicates that a strategy to elicit antibodies against this region may be an effective way of protecting against variants with amino acid changes in the RBD of the SARS-CoV-2 spike protein.

### CBM9-SARS-CoV-2 fusion proteins react with rabbit anti-spike protein sera and human sera

**Fig. 3:**
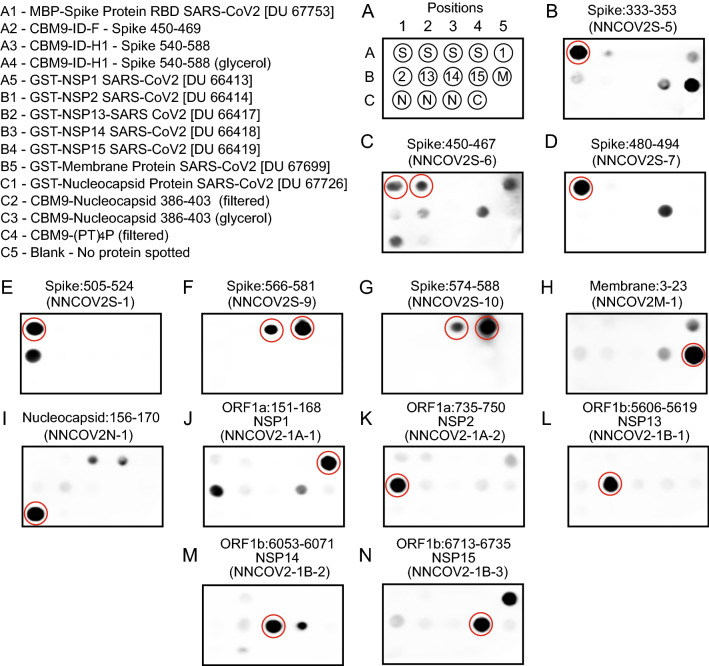
Dot blot analysis of SARS-CoV-2 recombinant proteins with rabbit polyclonal antibodies for diverse SARS-CoV-2 proteins. Expected target positions of SARS-CoV-2 proteins for each antibody are circled. Identification and location of each recombinant protein is shown in **A**

**Fig. 4:**
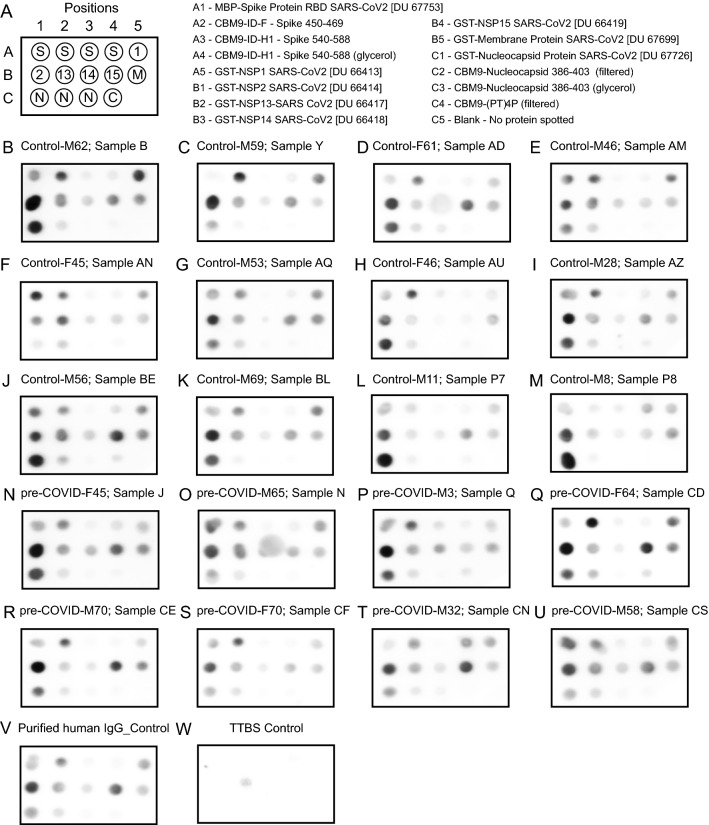
Dot blot of proteins with sera from pre-COVID and healthy controls. **B**–**M** “Control” samples correspond to healthy individuals whose serum samples were collected in 2020. **N**–**U** “pre-COVID” samples were from healthy individuals whose serum samples were retrieved prior to April 2019. Individuals are also identified by sex (M for male and F for female) followed by age in years. The MBP-spike RBD protein (spot position A1) includes amino acids 319–541 of the spike protein

**Fig. 5:**
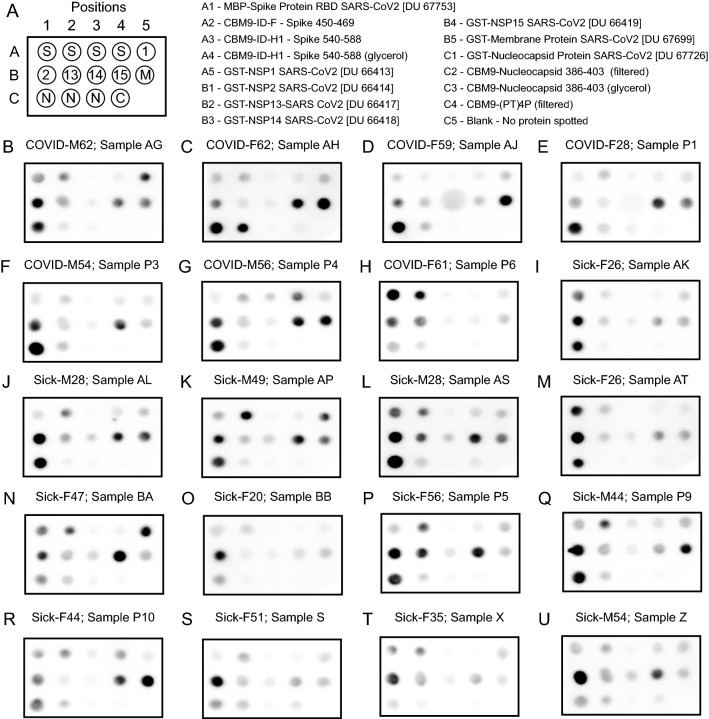
Dot blot of proteins with sera from COVID-19 and sick individuals. **B**–**H** “COVID” samples correspond to individuals that PCR-tested positive for SARS-CoV-2 RNA and whose serum samples were collected in 2020. **I**–**U** “Sick” samples were from individuals who had COVID-19 symptoms and whose serum samples were collected in 2020. Individuals are also identified by sex (M for male and F for female) followed by age in years. The MBP-spike RBD protein (spot position A1) includes amino acids 319–541 of the spike protein

As stated above, we used proline-threonine flexible linker regions to join the CBM9 module to SARS-CoV-2 spike protein and nucleocapsid protein regions. In this research the purpose of the linker was to allow the SARS-CoV-2 protein fragment to be accessible to antibody binding. To determine if the linker accomplished this, we reacted purified CBM9-(PT)_4_P, CBM9-ID-F, CBM9-ID-H1 and CBM9-N with purified rabbit antibodies that had been raised to different portions of SARS-CoV-2 proteins (Fig. 3) and human sera (Figs. 4, 5). In a semi-quantitative dot blot assay, we found that rabbit antibodies raised against the appropriate fragments of the SARS-CoV-2 spike protein reacted strongly with CBM9-H1 (Fig. 3F, G), but only weakly, or not at all, with antibodies directed against other regions of the spike protein. Reaction of the appropriate antibodies with CBM9-ID-F was moderately strong (Fig. 3C), and likewise poor or not detectable with the other antibodies. We had access to a small sampling of sera from COVID-19 confirmed (n = 7), COVID-19 suspected (n = 13), and healthy individuals (n = 20) (Figs. 4, 5). While this small sample set size and the dot blot assay cannot provide an epidemiological story, the results did show that human sera clearly reacted with the CBM9 fusions carrying ID-F and the nucleocapsid epitope. Many sera samples from both sick and healthy individuals reacted strongly with ID-F, indicating that this region may be similar in other coronaviruses or may be similar to another commonly encountered antigen. Surprisingly, the sera from several individuals, both healthy and ill, reacted apparently more strongly with CBM9-ID-F (spike amino acids 450–469) than with the MBP-RBD fusion protein (spike amino acids 319–541), even though the latter encompasses the ID-F region. These results may reflect a property of the antigen, such as accessibility of the SARS-CoV-2 portion to the antibody; or it may be that the ID-F region is an especially immunogenic region of the RBD. Overall, with this small sample of sera there was little difference between the patterns of reactivity of the sera from the sick and healthy groups. This high detection of anti-spike and anti-nucleocapsid immunoreactivity in serum samples from healthy individuals is consistent with previous studies using two different serological tests developed by Mesoscale Devices and Kinexus [40].

In this work, we have demonstrated that soluble fragments of the SARS-CoV-2 virus, fused to the CBM9 module through a flexible linker, could be produced at high levels—over 100 mg/L—using universally available equipment with inexpensive materials. The costs of cultivating *E. coli* are 10- to 100-fold less expensive than the costs of growing non-microbial eukaryotic cells, which are the usual hosts for expressing SARS-CoV-2 antigens. Further, the cost of using cellulose powder for affinity purification of CBM9 fusion proteins is about 100- to 1000-fold less than using the conventional immobilized nickel resin or a combination of traditional protein purification columns, such as ion-exchange with size exclusion resins. Lastly, while we described the production and isolation of CBM9 fusion proteins, there may be applications that require the separation of the CBM9 module from the SARS-CoV-2 fragment. Often the components of a fusion protein are separated using a highly specific protease cleavage site. Indeed, using this approach Kavoosi et al. [26] found that a CBM9-GFP fusion protein remained largely intact even in the absence of protease inhibitors, unless cleaved with factor Xa when a cleavage site was incorporated into the linker. Thus, the use of CBM9-SARS-CoV-2 protein fragment fusions allows for the economical production of antigens to be used for a variety of purposes, including in COVID-19 serological assays.

## Supplementary Information

**Additional file 1.** Proteomics methods, Source of commercial recombinant proteins, Sources of rabbit anti-peptide antibodies, Accession number of recombinant clones. **Table S1.** Primers used in this study. **Figure S1.** Example sequences of a CBM9-ID clone. **Figure S2.** SDS-PAGE of all recombinant clones. **Figure S3.** SDS-PAGE of CBM9-ID-H1 isolation and purification.

## Data Availability

All of the data relevant to the conclusion are presented in the manuscript or in the material presented in the additional file. The accession numbers for the nucleotide sequences of the recombinant clones and the source for obtaining clones is described in Additional file 1.
