# Supplementary material for: Escherichia coli recombinant expression of SARS-CoV-2 protein fragments
Source: Microb Cell Fact. 2022 Feb 5;21:21. doi: 10.1186/s12934-022-01753-0 (PMC8817660; doi:10.1186/s12934-022-01753-0)
Supplement: Supplementary file 1 — Additional file 1. Proteomics methods, Source of commercial recombinant proteins, Sources of rabbit anti-peptide antibodies, Accession number of recombinant clones. Table S1. Primers used in this study. Figure S1. Example sequences of a CBM9-ID clone. Figure S2. SDS-PAGE of all recombinant clones. Figure S3. SDS-PAGE of CBM9-ID-H1 isolation and purification. [file 12934_2022_1753_MOESM1_ESM.docx]

Additional File 1

*Escherichia coli* recombinant expression of SARS-CoV-2 protein fragments.

Bailey E. McGuire, Julia E. Mela, Vanessa C. Thompson, Logan R. Cucksey, Claire E. Stevens, Ralph L. McWhinnie, Dirk F. H. Winkler, Steven Pelech, and Francis E. Nano

Supplementary Materials and Methods.

Proteomics methods.

Commercial recombinant proteins.

Rabbit anti-peptide antibodies.

Accession number of recombinant clones.

Table S1. Primers used in this study

Figure S1. Example sequences of a CBM9-ID clone.

Figure S2. SDS-PAGE of all recombinant clones.

Figure S3. Fluorescence image of SDS-PAGE of isolation of CBM9-H1.

**Proteomics methods as provided by The University of Victoria Genome BC Proteomics Centre (**[**https://www.proteincentre.com/**](https://www.proteincentre.com/)**). Copied with permission.**

LC-MS analysis: Orbitrap Fusion

The protein samples (1 µL, ~0.5 µg) were separated by using a Waters Acquity UHPLC system (Milford, MA, USA) with a reversed-phase ACQUITY UPLC BEH C8 Column (1.7 µm, 130 Å, 1 mm x 150 mm) heated to 35 °C. The chromatography system was coupled on-line with an Orbitrap Fusion Tribrid mass spectrometer (Thermo Fisher Scientific, San Jose, CA) equipped with an EASY-Max NG ion source (Thermo Fisher Scientific). Solvents were A: 2% acetonitrile/0.1 % formic acid, B: 100% acetonitrile, 0.1% formic acid. After diverting the flow rate to waste for the first 2.5 min after injection the samples were separated using a 22-minute gradient (0 min: 5% B; 2 min: 10% B, 15 min: 85% B, 15.1 min: 90% B, 20 min: 90% B, 20.1 min: 5% B, 22 min: 5% B) at 100 µL min^-1^. The Orbitrap Fusion instrument parameters (Fusion Tune 3.3 software) were as follows for Orbitrap (OT-MS) mass spectrometry analysis: ion source spray voltage 3.5 kV, ion transfer tube temperature 275 ℃, S-lens RF level 60, vaporiser temperature 75 °C, sheath gas and auxiliary gas flow rates 25 and 5, respectively. The survey MS1 scan range 350-2000 m/z was acquired as profile mode data, resolution 120,000 FWHM@200m/z, one microscan with Auto maximum inject time. The Siloxane mass 391.28428 was used as lock mass for internal calibration. The automatic gain control (AGC) target values for FTMS was 100% (or 4e^5^).

Data Analysis Parameters

Raw files were created by Xcalibur 4.3.73.11 (Thermo Scientific) software and viewed using Freestyle Version software package 1.3.22.134 (Thermo Scientific) using the Xtract feature to obtain deconvoluted masses.

**Commercial recombinant proteins.**

MBP-Spike RBD amino acids (aa) 319-541 (DU 67753); GST-NSP1 aa 1-180 (DU 66413); GST-NSP2 aa 1-638 (DU 66414); NSP13 aa 1-601 (DU 66417); GST-NSP14 aa 1-527 (DU 66418); GST-NSP15 aa 1-527 (DU 66419); GST-Membrane Protein aa 1-222 (DU 67699); and GST-Nucleocapsid Protein aa 1-419 (DU 67726).

**Rabbit anti-peptide polyclonal antibodies.**

Spike aa 333-353 (NNCOV2S-5); Spike aa 450-467 (NNCOV2S-6); Spike aa 480-494 (NNCOV2S-7); Spike aa 505-524 (NNCOV2S-1); Spike aa 566-581 (NNCOV2S-9); Spike aa 574-588 (NNCOV2S-10); Membrane aa 3-23 (NNCOV2M-1); Nucleocapsid aa 156-170 (NNCOV2N-1); ORF1a aa 151-168 – NSP1 (NNCOV2-1A-1); ORF1a aa 735-750 – NSP2 (NNCOV2-1A-2); ORF1b aa 5606-5619 – NSP13 (NNCOV2-1B-1); ORF1b aa 6053-6071 – NSP14 (NNCOV-1B-2) and ORF1b aa 6713-6735 – NSP15 (NNCOV2-1B-3).

**Accession numbers and availability of recombinant clones.** The nucleotide sequences for all of the clones described in this work were deposited with GenBank (CBM9-(PT)_4_P, MZ322548; CBM9-ID-A, MZ322549; CBM9-ID-B, MZ322550; CBM9-ID-C, MZ322551; CBM9-ID-D, MZ322552; CBM9-ID-E, MZ322553; CBM9-ID-F, MZ322554; CBM9-ID-G, MZ322555; CBM9-ID-H1, MZ322556; CBM9-N, MZ322557). The recombinant clones expressing CBM9-(PT)_4_P, CBM9-ID-F, CBM9-ID-H1 and CBM9-N have been deposited with AddGene (<https://www.addgene.org/>).

Table S1. Primers used in this study

| Primer Name | Purpose or sequence of primers  Red text indicates overhang regions generated by cutting with Esp3I or BsaI |
| --- | --- |
|  |  |
|  | Primers for inverse PCR of pRSET5A for cloning synthetic DNA encoding CBM9-ID-A, C and H3. |
|  |  |
| F-R5A | CGTCTCaCCTTCCGCTGAGCAATAACTAGCATAAC |
|  |  |
| R-R5A | CGTCTCaACCACCAGTCATGCTAGCCA |
|  |  |
|  | Primers for amplifying synthetic DNA for nCBM9 (native DNA sequence) for cloning into pRSET5A iPCR amplicon. |
|  |  |
| F-nCBD | GGTCTCaTGGTAGCGGAATAATGGTAGCGACAG |
|  |  |
| R-nCBD | GGTCTCaAAGGGCTTCACTTGATGAGCCTGAGG |
|  |  |
|  | Primers for amplifying synthetic DNA for CBM9-ID-C, F, and H3 for cloning into pRSET5A iPCR amplicon. |
|  |  |
| F-CBD | CGTCTCaTGGTAGCGGTATCATGGTTGCTACC |
|  |  |
| R-CBD-IDc | CGTCTCaAAGGGCTTCATTAGTCAACAGCGTCGG |
|  |  |
| R-CBD-IDf | CGTCTCaAAGGGCTTCATTACTGGTAGATTTCGGTAGAGATG |
|  |  |
| R-CBD-H3 | CGTCTCaAAGGGCTTCATTAGTCACGAACAGCGTC |
|  |  |
|  | Primers for iPCR of pRSET5A::*CBM9-id-c* to remove ID-C region and insert epitopes ID-A, B, D, E, G, H, H1, H2, and I. |
|  |  |
| Fb-R5A | attCGTCTCaCCTTCCGCTGAGCAATAACTAGCATAAC |
|  |  |
| Rb-R5AidC | attCGTCTCaTCAGACGCAGGTTACCGAATTTAGACG |
|  |  |
|  | Primers for iPCR of pRSET5A::*CBM9-id-a* to eliminate ID-A region to generate clone expressing CBM9-(PT)_4_P. |
|  |  |
| nF2-R5A-CBD | TTACGTCTCAAATGCGATGTACGTGCTATGCTCC |
|  |  |
| nR-R5A-Flex | TTACGTCTCACATTTCATTACGGTGTTGGGGTAGGTGT |
|  |  |
|  | Primers for iPCR of pRSET5A::*CBM9-id-a* to eliminate ID-A region to generate clone expressing a nucleocapsid epitope. |
|  |  |
| F-nucl-ep | aatCGTCTCaGCCGGCTGCTGACCTGGACGACTTCTAATGACTTGCGATGTACGTGCTATGCT |
|  |  |
| R-nucl-ep | aatCGTCTCaCGGCAGCAGGGTAACGGTCTGCTGTTTTTTCTGCGGTGTTGGGGTAGGTGTTG |
|  |  |
|  | Primers for amplifying region encoding CBM9 fusions for sequencing and use in sequencing reactions. |
|  |  |
| nFseq5A | GATCTCGATCCCGCGAAATTAATAC |
|  |  |
| nRseq5A | aaacccctcaagacccg |
|  |  |

**>CBM9-ID-A**

ACTTTAAGAAGGAGATATACAT**ATG**GCTAGCATGACTGGTGGTAGCGGTATCATGGTTGCTACCGCTAAATACGGTACCCCGGTTATCGACGGTGAAATCGACGAAATCTGGAACACCACCGAAGAAATCGAAACCAAAGCTGTTGCTATGGGTTCTCTGGACAAAAACGCTACCGCTAAAGTTCGTGTTCTGTGGGACGAAAACTACCTGTACGTTCTGGCTATCGTTAAAGACCCGGTTCTGAACAAAGACAACTCTAACCCGTGGGAACAGGACTCTGTTGAAATCTTCATCGACGAAAACAACCACAAAACCGGTTACTACGAAGACGACGACGCTCAGTTCCGTGTTAACTACATGAACGAACAGACCTTCGGTACCGGTGGTTCTCCGGCTCGTTTCAAAACCGCTGTTAAACTGATCGAAGGTGGTTACATCGTTGAAGCTGCTATCAAATGGAAAACCATCAAACCGACCCCGAACACCGTTATCGGTTTCAACATCCAGGTTAACGACGCTAACGAAAAAGGTCAGCGTGTTGGTATCATCTCTTGGTCTGACCCGACCAACAACTCTTGGCGTGACCCGTCTAAATTCGGTAACCTGCGTCTGATCAAACCGACGCCAACACCTACCCCAACACCGAACCTGACCACCCGTACCCAGCTGCCGCCGGCTTACACCAACTCTTTCACCCGTGGTGTTTACTACCCGGACAAAGTTTTCCGTTCTTCTGTTCTGCACTCTACCCAG**TAATGA**CTTGCGATGTACGTGCTATGCT

>CBM9-ID-A protein, MW 27,220

MASMTGGSGIMVATAKYGTPVIDGEIDEIWNTTEEIETKAVAMGSLDKNATAKVRVLWDENYLYVLAIVKDPVLNKDNSNPWEQDSVEIFIDENNHKTGYYEDDDAQFRVNYMNEQTFGTGGSPARFKTAVKLIEGGYIVEAAIKWKTIKPTPNTVIGFNIQVNDANEKGQRVGIISWSDPTNNSWRDPSKFGNLRLIKPTPTPTPTPNLTTRTQLPPAYTNSFTRGVYYPDKVFRSSVLHSTQ

Figure S1. Example sequence of a CBM9-ID clone. Red: plasmid sequence. **Red/bold**: start codon. Black: CBM9. Blue: (PT)_4_P. Brown: ID-A sequence. **Black/bold**: stop codons. Green: random DNA sequence. All of the CBM9 fusions have been deposited at GenBank (see Materials and Methods).


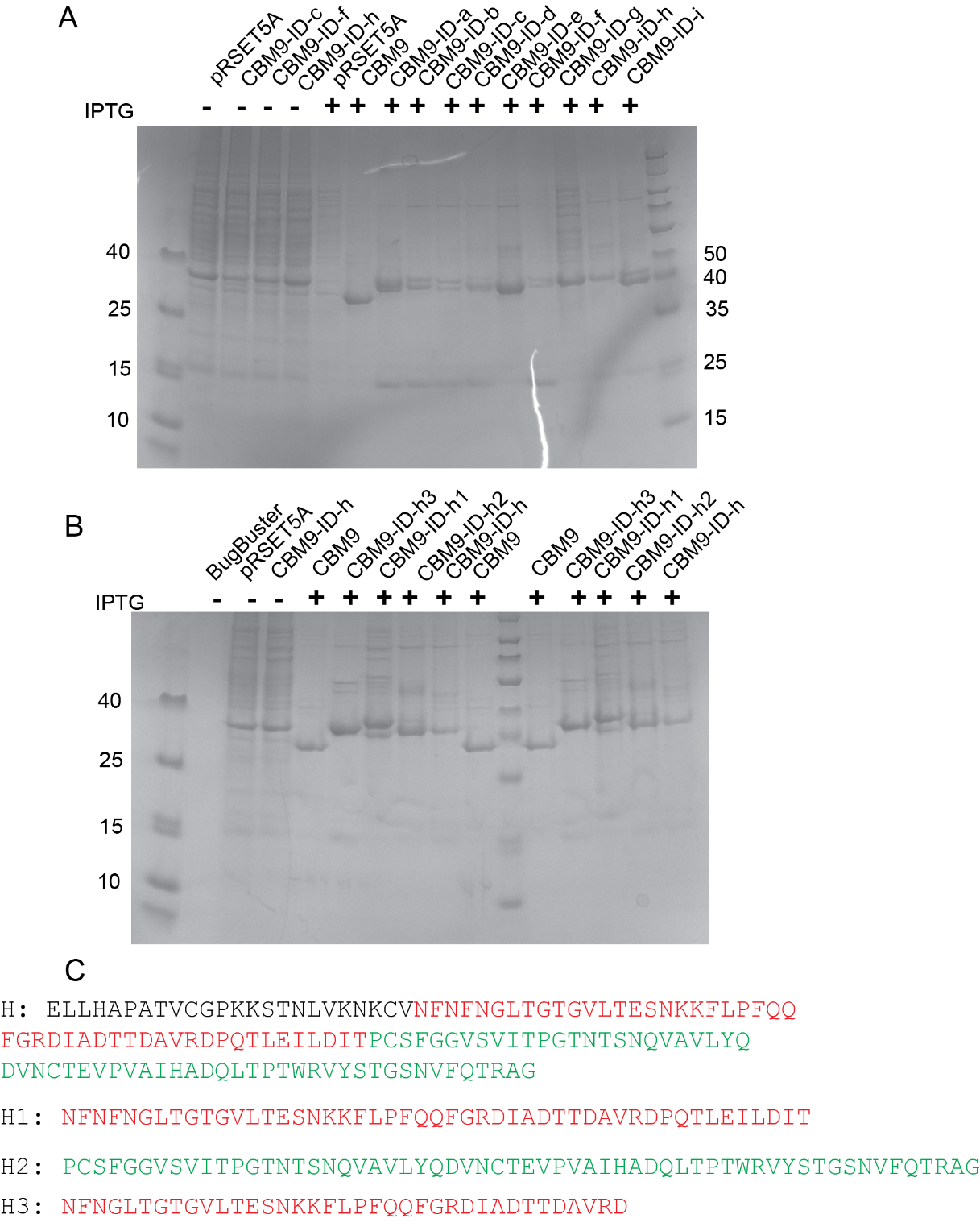


Figure S2. Expression of recombinant CBM9 fusion proteins in *E coli*. A and B. SDS-PAGE gels of *E. coli* cell lysates from cells expressing recombinant CBM9 fusion proteins, in the presence and absence of IPTG inducer. C. Amino acid sequences of ID-H, H1, H2 and H3 regions. Various colored fonts are used to depict different segments of the ID-H region. Position of molecular weight markers are shown on the side of the gel.


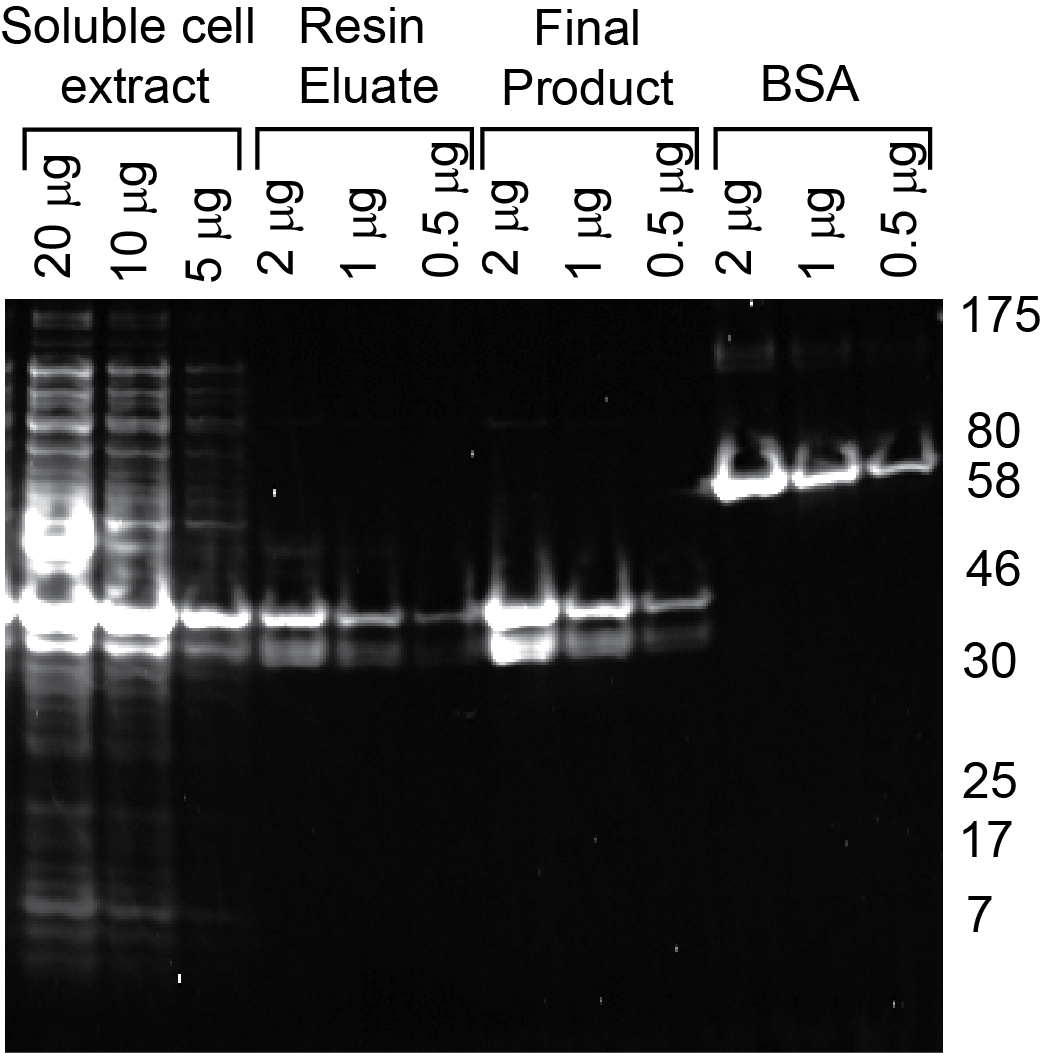


Figure S3. Preparative isolation of CBM9-ID-H1 using batch purification with cellulose resin. Proteins were separated by 12% SDS-PAGE, stained with Coomassie blue, and a LI-COR Odyssey® CLx Imager was used to scan fluorescence of Coomassie-stained protein bands excited at 700 nm. Position of molecular weight markers are shown to the right of the gel.
